# Analysis of Amyloid Fibrillation of Two Family 1 Glycoside Hydrolases

**DOI:** 10.3390/ijms25158536

**Published:** 2024-08-05

**Authors:** Miguel A. Rodríguez-López, José María Coll-Marqués, David Talens-Perales, Julia Marín-Navarro, Julio Polaina, Edgar Vázquez-Contreras

**Affiliations:** 1 Postgraduate in Natural Sciences and Engineering, Autonomous Metropolitan University, Cuajimalpa, Mexico City 05348, Mexico; miguel.rodriguez.l@cua.uam.mx; 2 Departament of Natural Sciences, Autonomous Metropolitan University, Cuajimalpa, Mexico City 05348, Mexico; 3 Institute of Agrochemistry and Food Technology, Spanish National Research Council (IATA-CSIC), 46980 Paterna, Valencia, Spain; jcoll@iata.csic.es (J.M.C.-M.); d.talens@iata.csic.es (D.T.-P.);; 4 Departament of Biochemistry and Molecular Biology, University of Valencia, 46100 Burjassot, Valencia, Spain

**Keywords:** β-glucosidase, TIM barrel, protein structure

## Abstract

The formation and analysis of amyloid fibers by two β-glucosidases, BglA and BglB, belonging to the GH1 enzyme family, are reported. Both proteins have the (β/α)_8_ TIM-barrel fold, which is characteristic of this family and is also the most common protein structure. BglA is an octamer, whereas BglB is a monomer. Amyloid fibrillation using pH and temperature as perturbing agents was investigated using fluorescence spectroscopy as a preliminary approach and corroborated using wide-field optical microscopy, confocal microscopy, and field-emission scanning electron microscopy. These analyses showed that both enzymes fibrillate at a wide range of acidic and alkaline conditions and at several temperature conditions, particularly at acidic pH (3–4) and at temperatures between 45 and 65 °C. Circular dichroism spectroscopy corroborated the transition from an α-helix to a β-sheet secondary structure of both proteins in conditions where fibrillation was observed. Overall, our results suggest that fibrillation is a rather common phenomenon caused by protein misfolding, driven by a transition from an α-helix to a β-sheet secondary structure, that many proteins can undergo if subjected to conditions that disturb their native conformation.

## 1. Introduction

Amyloidoses are a type of deposition disease characterized by the formation and accumulation of protein complexes. They are generated by the coalescence of unfolded protein molecules that grow to form an orderly braid-shaped pattern. Amyloidoses include some of the most threatening human ailments because of their growing incidence, particularly among elderly people, such as type 2 diabetes, Alzheimer’s and Parkinson’s diseases, and many other disorders affecting heart, liver, kidneys, and virtually every other organ [1,2]. Whereas proteins with different structure can be precursors of the aggregates that give rise to deposition diseases, in all cases, the formation of fibrillar aggregates occurs by the adhesion of β-strand protein segments into β-sheet structures, which is considered a critical event at the onset of the pathological process [2,3,4,5,6]. Amyloid plaques, characteristically observed in the analysis of tissues affected by neurodegenerative diseases, are formed by the accumulation of protein fibrils [7,8,9,10,11].

In vitro analysis carried out with different proteins involved in amyloidoses constitutes a powerful approach to decipher the molecular mechanism of fibrillogenesis. These studies [12,13,14] eventually should lead to the discovery of molecules that inhibit the process and help design therapies against protein deposition diseases. We have studied the formation of amyloid fibers using two enzymes belonging to the glycoside hydrolase family 1 (GHF1). Proteins of this family are characterized by having a TIM-barrel structure, the most common structure cataloged in the Protein Data Bank [15]. Outstanding members of the GHF1 are the non-enzymatic Klotho proteins, whose deficiency has been found to be a determinant factor in human aging [16,17,18]. Another relevant member of this family is the human intestinal lactase-phlorizin hydrolase (LPH), whose deficiency causes lactose intolerance [19]. The two enzymes used here (BglA and BglB) are β-glucosidases isolated from the bacterium *Paenibacillus polymyxa*. They differ in their quaternary structure: BglA is as an octamer, whereas BglB is a monomer [20,21,22,23]. BglA and BglB were chosen for two reasons. Firstly, their common 3D structure (TIM barrel) makes them suitable representatives of the average protein. Secondly, their enzymatic activity can be measured very easily, which is a very convenient property to correlate the structure and functional state of a protein. As a first approach, structural changes that could result in fibrillation were induced by changing pH and temperature conditions and then monitored using fluorescence spectroscopy. Extrinsic fluorescence upon protein binding to thioflavin T (ThT) and 8-anilino-1-naphthalenesulfonic acid (ANS) fluorophores as well as intrinsic fluorescence and enzyme activity were measured. The formation of fibers was further characterized by optical and field-emission scanning electron microscopy. The results obtained supply relevant data relating to protein structure, amyloid fibrillation, and function.

## 2. Results and Discussion

### 2.1. Amylogenic Regions in BglA and BglB Structures

**Figure 1:**
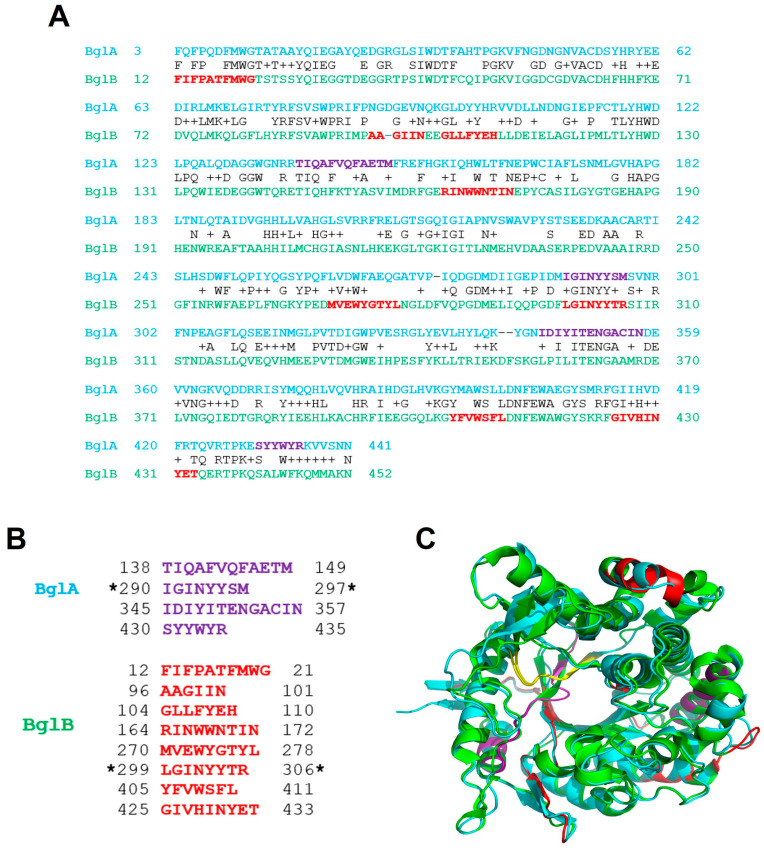
Protein structure of BglA and BglB and mapping of the predicted amylogenic regions. (**A**) Alignment of BglA and BglB amino acid sequences. Predicted amylogenic regions in BglA and BglB are depicted in purple and red, respectively. (**B**) Sequences of predicted amylogenic regions in the BglA and BglB primary structures. Asterisks indicate the amylogenic region shared by both proteins. (**C**) Superimposed three-dimensional structure of a BglA monomer (cyan) and BglB (green), with PDB codes 1BGA and 2O9P, respectively. The color code is the same one used in panel (**A**), except the yellow region corresponds to the predicted amylogenic region shared by BglA and BglB.

BglA and BglB were taken as representative examples of the most common protein structure, the TIM barrel [15]. The amino acid sequence of both proteins was analyzed using Waltz, an algorithm that predicts the existence of amylogenic regions in proteins based on the input of a large number of amylogenic peptides whose properties have been characterized experimentally [24,25]. It is becoming increasingly clear that amyloid fibrillogenesis is not restricted to a number of polypeptides associated with certain diseases but rather is a common feature of proteins determined by the presence of short hydrophobic stretches of amino acids [4,26,27,28,29]. Figure 1A shows the location of these regions, depicted in purple and red for BglA and BglB, respectively. Figure 1B summarizes the composition of the four potentially amylogenic sequence segments present in BglA and the eight in BglB. Figure 1C displays the superposition of the three-dimensional structures of the proteins, with the positions of the regions highlighted in the same colors used in panel A. Two of the BglA amylogenic regions are situated within the β-core of the TIM-barrel structure, involving loops and β-sheets, while the other two regions are in peripheral α-helices. For BglB, two regions appear in hydrophilic α-helices, whereas the others are in loops and β-sheets mostly in the interior of the barrel. Despite the close similarity between BglA and BglB, the regions predicted by Waltz are not found in the same positions, as shown in Figure 1C. There is only one predicted amylogenic region that is shared between both proteins, and it is located in their β-sheet core (shown in yellow in Figure 1C), spanning from residues 290 to 297 in BglA and from residues 299 to 306 in BglB (indicated by asterisks in Figure 1B). This suggests that despite their similarity in sequence and three-dimensional structure, BglA and BglB might have differences in their amylogenic properties.

### 2.2. Fluorometric and Microscopic Analysis of BglA and BglB Solutions at a Wide Range of pH and Temperature Conditions

**Figure 2:**
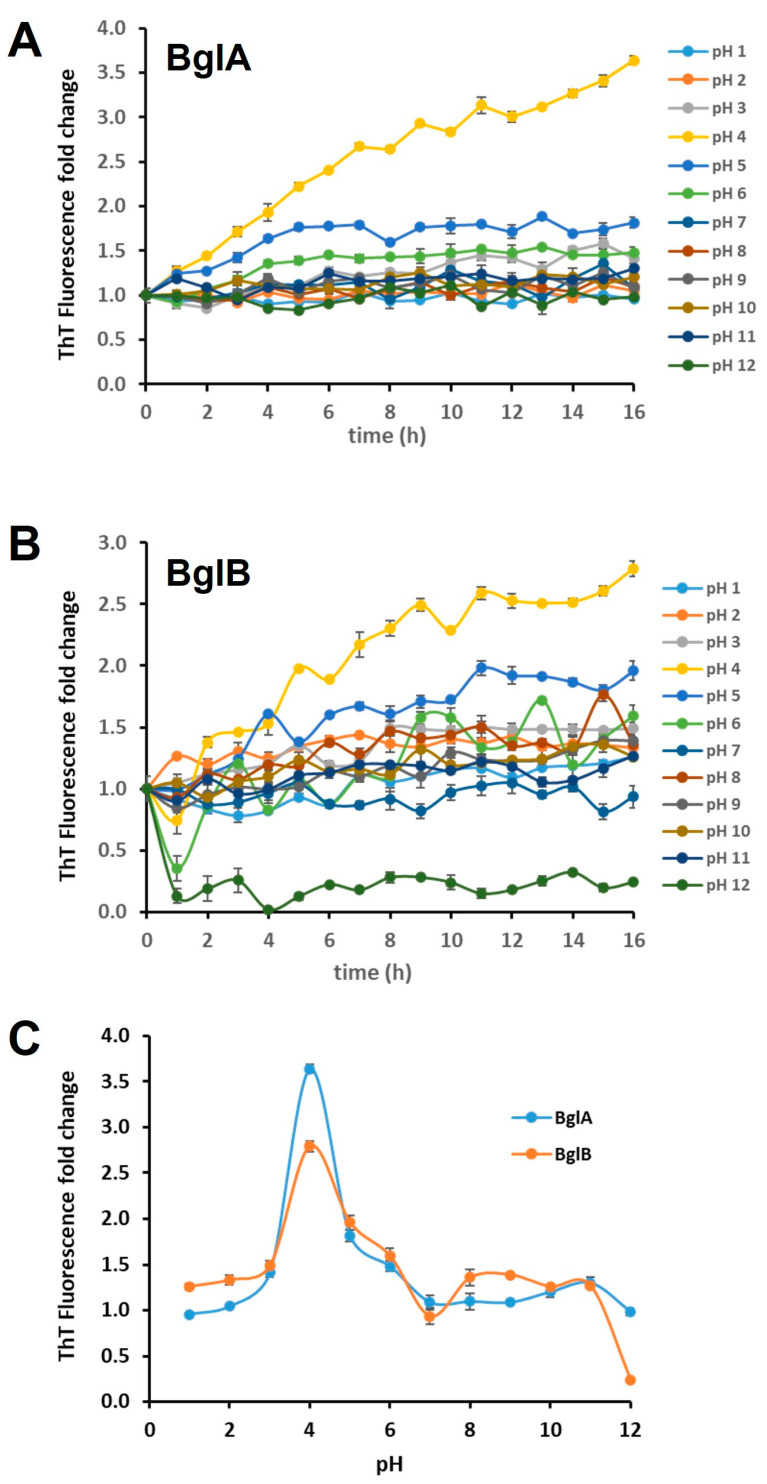
Fluorescence emission of thioflavin T (ThT) assay for BglA (panel (**A**)) and BglB (panel (**B**)) at different pH values. (**C**) ThT fluorescence after 16 h of incubation of BglA or BglB at different pH values. In all cases, ThT fluorescence intensity at 485 nm after excitation at 450 nm at time zero was assigned as the unit. All data are the average of three independent quantifications, and the standard deviation was less than 10% in all cases.

Changes in pH and temperature affect protein conformation and hence have an impact on amyloid fibril formation [30]. ThT fluorescence is commonly used for detecting and quantifying amyloid fibrils [31,32]. Therefore, we monitored fluorescence upon the addition of ThT to BglA and BglB as an indication of fibrillation for these proteins. The results shown in Figure 2 show an increase in fluorescence after a few hours of incubation at both acidic and alkaline pH values for both proteins at a concentration of 1 mgmL^−1^. This suggests that they form amyloid structures, which is in agreement with the presence of amyloid regions in their sequences. In both cases, a maximum value of fluorescence is observed at a pH of 4.

**Figure 3:**
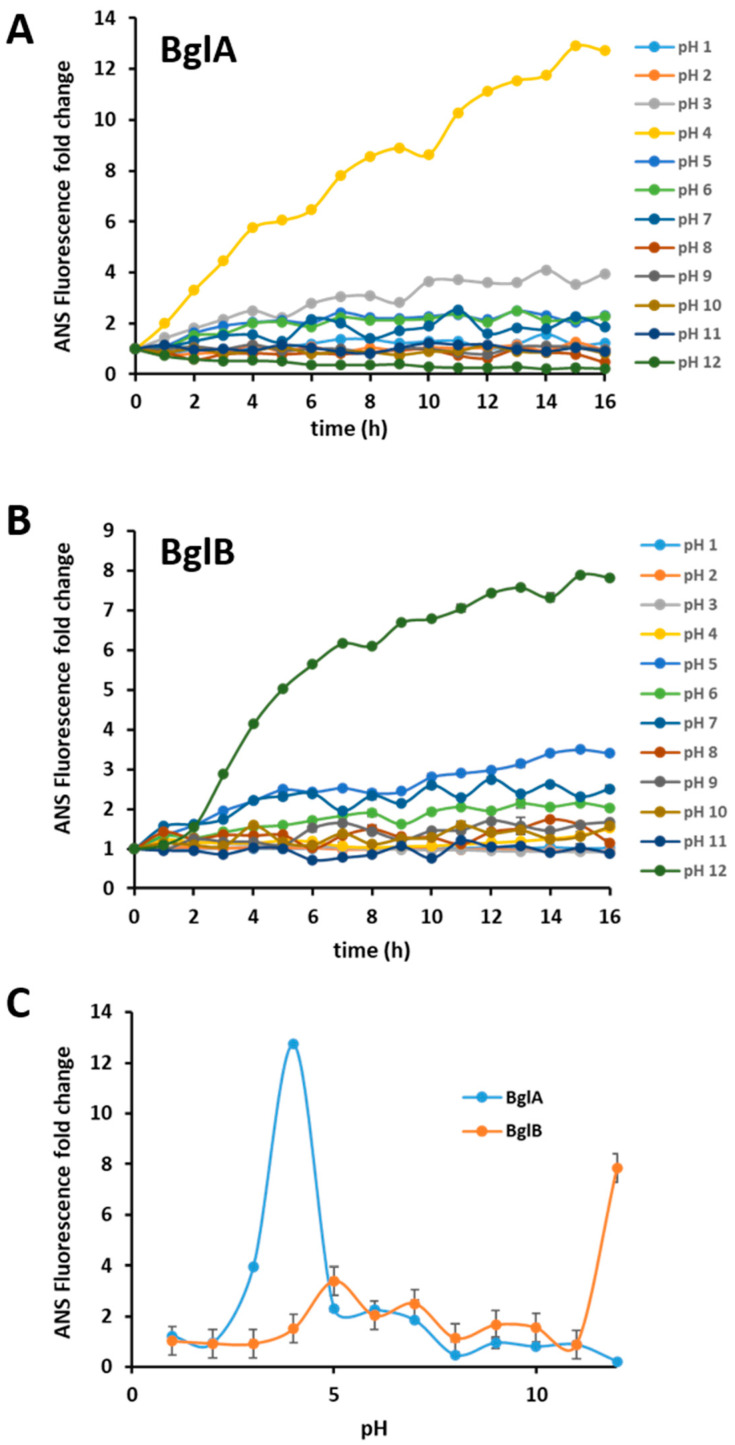
Fluorescence emission of 8-anilino-1-naphthalenesulfonic acid (ANS) assay for BglA (panel (**A**)) and BglB (panel (**B**)) at different pH values. (**C**) ANS fluorescence after 16 h of incubation of BglA or BglB at different pH values. In all cases, ANS fluorescence intensity at 470 nm, after excitation at 380 nm at time zero, was assigned as the unit. All the data are the averages of three independent measurements, and the standard deviation was less than 10% in all cases.

As a complementary approach to monitor structural changes of the protein structure, we used ANS, another fluorophore frequently employed for studying protein folding and other questions related to protein structure, including the formation of amyloid fibers [33,34]. Similarly to what was observed for ThT, the results depicted in Figure 3 show an increase in ANS fluorescence for BglA and BglB at both acidic and alkaline pH values. However, for ANS, the fluorescence profiles of the two proteins are quite different (Figure 3C), with BglA showing a sharp increase at a pH of around 4. This likely reflects the main structural difference between the two proteins, which is their quaternary structure, as BglA is an octamer whereas BglB is a monomer. In oligomeric proteins, ANS binds with high affinity to hydrophobic regions that maintain the quaternary structure when they become exposed by partial unfolding, whereas it shows little interaction with fully unfolded proteins.

A further approach to investigate changes in the protein structure and its connection with amyloid fibrillation was measuring the intrinsic and extrinsic protein fluorescence, as well as residual catalytic activity, at different values of temperature and pH. Two procedures were used to measure intrinsic fluorescence. In the first method, the sample was irradiated at 280 nm, which excites all intrinsic fluorophores: tryptophan, tyrosine, and phenylalanine. In the second approach, the excitation wavelength was 295 nm, which selectively targets tryptophan residues. BglA and BglB are not ideal models for these studies due to their high number of aromatic residues. BglA has 61 aromatic residues while BglB has 59, which for both proteins constitute more than 13% of their respective amino acid content. Nevertheless, they still can provide useful information regarding major changes in the protein three-dimensional structure. Extrinsic fluorescence, with the same two fluorophores utilized in previous analyses, ThT and ANS, was also measured. Finally, the native, functional state of the enzymes was assessed by measuring their catalytic activity using p-nitrophenyl-β-D-glucopyranoside as the substrate. Table 1 shows the overall result of these experiments. Both proteins, BglA and BglB, remain stable at pH 7.0 and 25 ºC without significant changes in intrinsic or extrinsic florescence or enzyme activity. In general terms, all treatments departing from these conditions that represent a “comfort zone” for the proteins imply some degree of unfolding of the native structure, as indicated by changes in intrinsic or ANS fluorescence. When protein fibrillation was monitored with the ThT probe, the highest values were observed at pH 3 for both proteins. In the case of BglA, such an increase was similar at 25 or 65 °C. Surprisingly, at 45 °C, ThT fluorescence was reduced after 16 h of incubation. This may be caused by a partial, transitory denaturation of the enzyme that releases ThT bound to native β-structures (see below). For BglB, at pH 3, ThT fluorescence increased sharply at 45 and 65, being higher at 45 °C. At pH 7, increased ThT fluorescence was only observed for BglB at 45 and 65 °C. At pH 12, no increase in ThT fluorescence for either of the two proteins was observed. Remarkably, ThT fluorescence increase, henceforth fibrillation, in some conditions, i.e., pH 3, 25 °C for BglA and BglB; and pH 7, 45 °C for BglB, was not associated with a substantial loss of catalytic activity.

Hen egg white lysozyme (HEWL), a glycoside hydrolase belonging to the GH22 family, has been extensively used to study protein fibrillation [35]. The enzymes used in this study constitute a suitable alternative model system. The simplicity of their enzyme assay offers a robust method to monitor the functional state of the protein. Furthermore, the transitions of these enzymes into the amyloid state can be utilized for screening compounds that have the potential to interfere with or even reverse this process. This opens possibilities for identifying compounds that can modulate amyloid formation and potentially serve as therapeutic agents.

**Figure 4:**
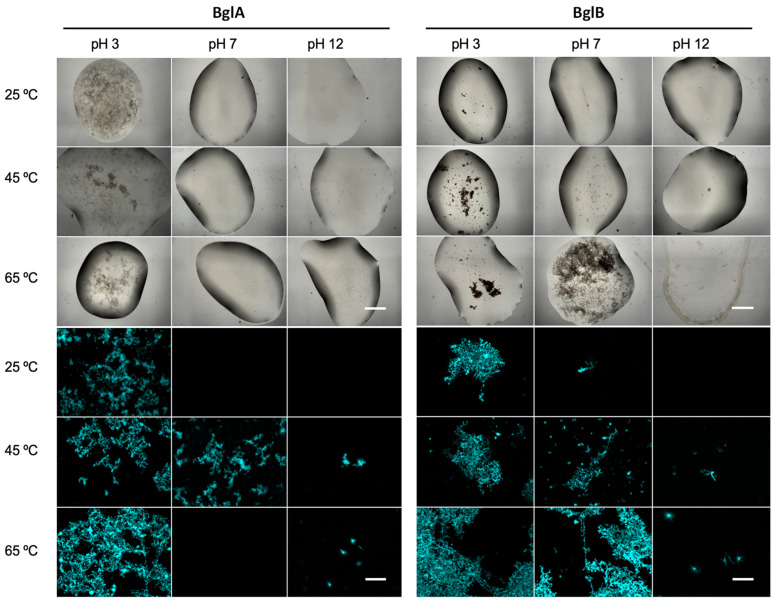
Wide-field optical microscopy of BglA and BglB protein aggregates after 8 days of incubation using either bright-field or epifluorescence illumination. The pictures in the upper half of the figure were imaged with bright-field illumination using a 2× objective lens combined with an optical zoom factor of 0.8. Scale bar, 1 mm. The pictures in the bottom half of the picture were taken with an epifluorescence illumination of the same samples, stained with ThT. A 40× objective lens combined with an optical zoom factor of 0.8 was used. Scale bar, 50 µm.

To establish a possible correlation between increased fluorescence and fibrillation, protein samples (1 mgmL^−1^) were incubated under the same pH and temperature conditions as in Table 1 and observed under the microscope after different incubation times. Bright-field illumination and ThT extrinsic fluorescence were used for the analysis. Figure 4 shows the results observed after 8 days of incubation. Some of the samples analyzed that showed protein aggregation under bright-field illumination displayed fibrillar structures when observed with ThT fluorescence. At pH 3, protein aggregates, reminiscent of amyloid plaques, appeared in BglA and BglB at all three tested temperatures, although fewer aggregates were observed at 25 °C. Unexpected ThT fluorescence decrease observed at 45 °C after 16 h for BglA (Table 1) should be a transient phase, as abundant fibrillation was observed at a later stage (8 days, Figure 4). At pH 7, BglA yielded “amyloid plaques” only at 45 °C, whereas for BglB, these aggregates were observed at 45 and 65 °C. No such structures appeared at pH 12.

**Figure 5:**
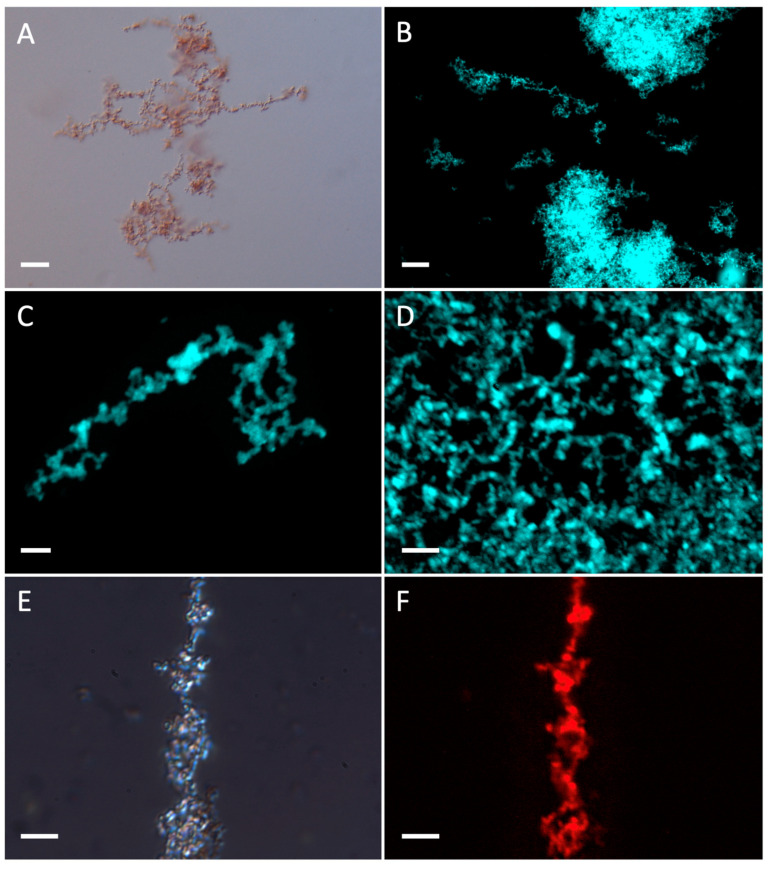
Wide-field optical microscopy of BglB and BglA protein aggregates using either differential interference contrast microscopy Nomarsky (DIC/N) or epifluorescence illumination after ThT or Congo red staining. Panel (**A**): BglA, pH 7.0, 65 °C (DIC/N). Panels (**B**–**D**): BglB, pH 3.0, 45 °C (ThT). Panel (**E**): BglB, pH 3.0, 45 °C (DIC/N). Panel (**F**): BglB, pH 3.0, 45 °C (CR). Scale bar: 20 µm in panels (**A**,**B**); 5 µm in panels (**C**–**F**).

Figure 5 shows the results of a more detailed analysis of the protein precipitates observed in Figure 4. For this analysis, the proteins were stained with ThT and Congo red, two dyes that specifically stain amyloid fibers. Microscopic observation, which was carried out at higher magnification, demonstrated that the glucosidases yielded fibrillar structures. Congo red staining and subsequent observation under polarized light, revealed birefringence in the samples, as it is characteristic for amyloid fibers. The birefringent colors observed were blue and red. Although it is generally assumed that the green color is crucial for diagnosing amyloid structures, it has been clearly shown that Congo red staining can result in other colors [36,37,38].

### 2.3. FESEM and Confocal Microscopy Analysis of Amyloid Fibers

**Figure 6:**
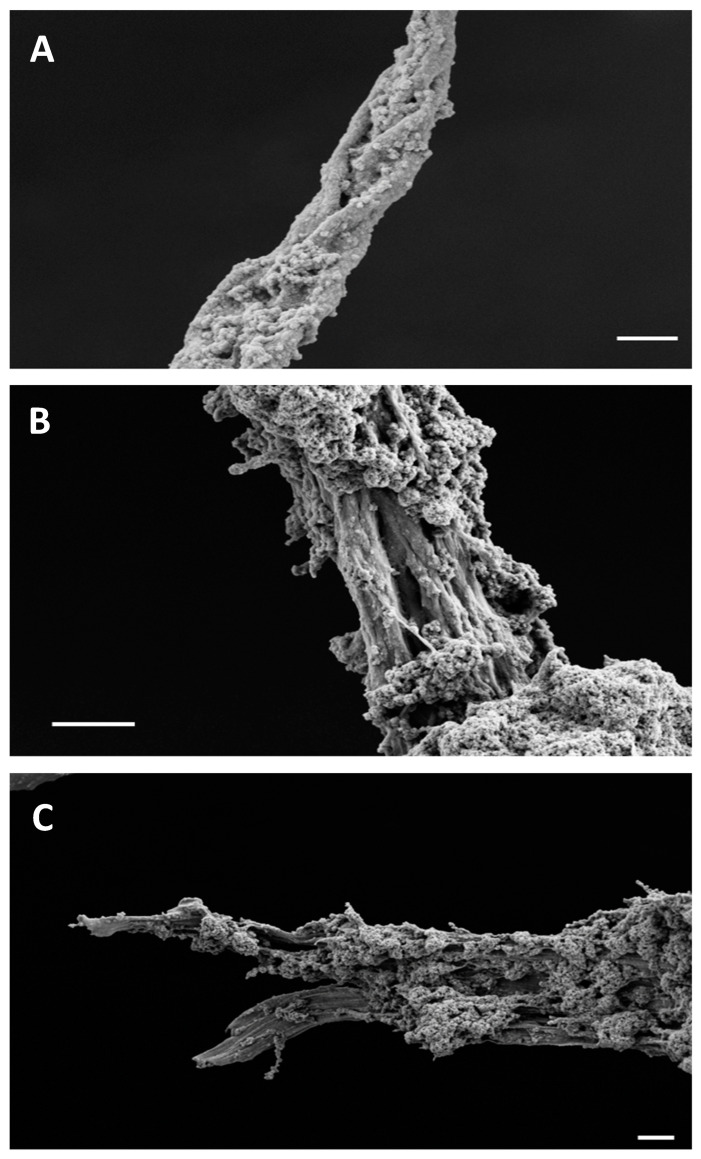
Scanning electron microscopy (FESEM) pictures of amyloid fibers of BglB (panels (**A**,**B**)) and BglA (panel (**C**)). Samples of protein (1 mgmL^−1^) were incubated at pH 3 and room temperature for 96 h. Scale bar: 1 µm (panel (**A**)), 2 µm (panels (**B**,**C**)).

The detailed morphology of the amyloid structures formed by BglA and BglB was investigated by field-emission scanning electron microscopy (FESEM). This procedure has been used in the study of these kinds of structures and provides valuable insights into their morphology and aggregation properties [39]. Figure 6A shows a BglB superstructure in which tightly packed protofilament bundles of around 200–300 nm each intertwine with each other, forming a braid-like structure with a thickness of approximately 1 µm. Each of these bundles is likely the result of the association of hundreds of protein protofilaments. Figure 6B illustrates another BglB superstructure formed by the coalescence of several 1 µm filaments. Intermediate 200 nm filaments are also visible. Figure 6C displays a view of the distal region of a mature superstructure of BglA protein. Amyloid fibrils have been reported to be generally composed by 2–6 protofilaments, showing diameters ranging from 5 to 15 nm, although fibrils with more than 100 protofilaments and a diameter of up to 150 nm have also been described [11,40]. FESEM observations reported here show very large braid-like structures with a thickness of 1 µm. These braids are formed by clearly visible fibers with a diameter of around 200–300 nm each, which in turn are likely composed by hundreds of tightly packed protofibrils. Thus, the large braid-like structures represent a superior degree of structure, comparable with what has been described for insulin upon multiple rounds of self-seeding [41]. The precise atomic arrangement of these structures can be revealed by CryoEM analysis [42].

**Figure 7:**
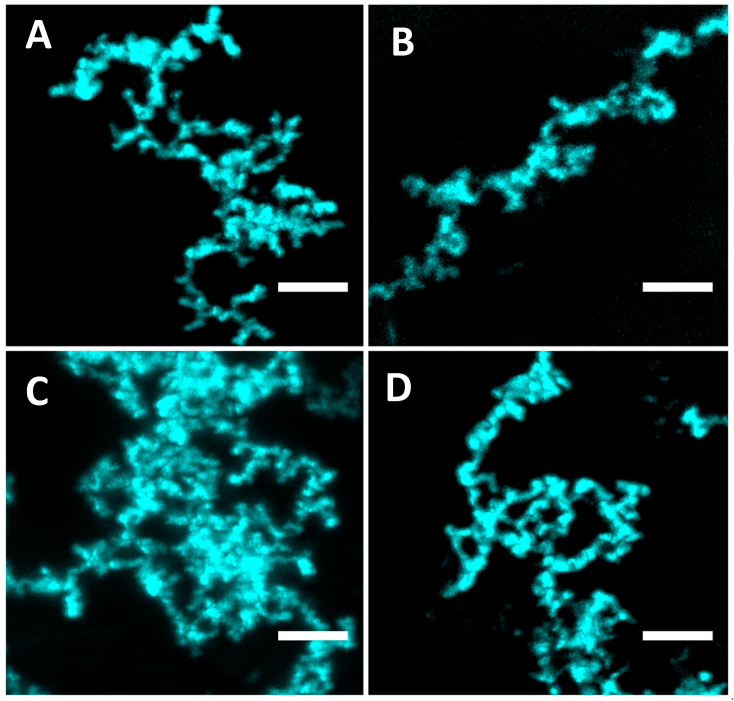
Confocal microscopy of ThT-stained BglA (panels (**A**,**B**)) and BglB (panels (**C**,**D**)) protein aggregates. Images are the maximum projection of each Z-stack. Field of view: 26 × 26 µm. Z-step = 0.15 µm. Total depth: 2.7 µm (18 slices); panel (**A**): 8.3 µm (55 slices); panel (**B**): 9.0 µm (60 slices); panel (**C**): 8.7 µm (58 slices); panel (**D**): illumination: 405 nm laser.

Finally, confocal microscopy was also used to analyze the fibrillar material obtained with BglA and BglB samples, incubated under the conditions described for Figure 4 and stained with ThT. The results shown in Figure 7 allowed us to visualize an uninterrupted thread that clearly shows the expected properties of an amyloid fiber.

### 2.4. Circular Dichroism Spectroscopy Analysis

**Figure 8:**
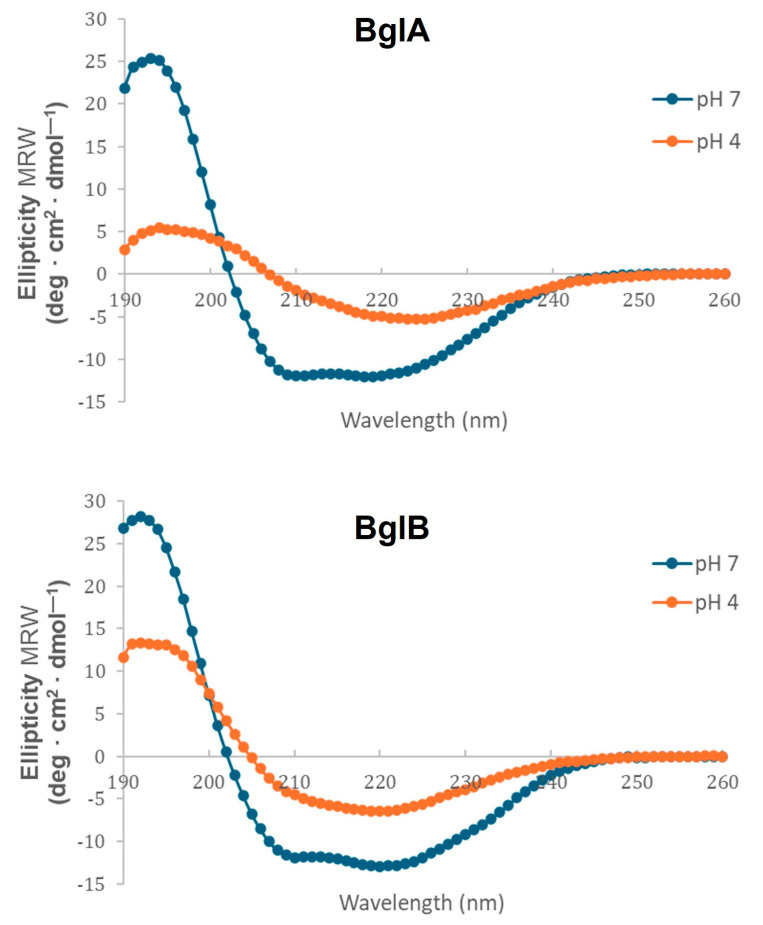
Far-UV CD spectra of BglA and BglB recorded after 16 h of incubation at 25 °C and pH 7 or 4.

The results of the analysis of fluorescence emission mediated by the binding of ThT to BglA and BglB (Figure 2) suggested that both proteins could fibrillate under different conditions of pH. Moreover, fibril formation seemed to be favored at a pH of around 4. This observation was subsequently confirmed by both light and electron microscopy. At the molecular level, protein amyloid fibrillation is characterized in all cases by the formation of a β-sheet structure, which is preceded by a transition of the secondary structure elements of the protein involved from an α-helix to a β-strand [2,3,4,5,6]. This transition can be best analyzed by CD and is revealed by a shift in the spectrum with an increasing intensity of the β-sheet signal, showing a negative peak around 218 nm, the so called “β-sheet CD signature”. Therefore, we carried out a CD analysis of BglA and BglB, comparing the spectra of the proteins in their native state (25 °C, pH 7) with the conditions at which the ThT peak was observed (25 °C, pH 4). The results presented in Figure 8 clearly show the expected sharp transition to a β-sheet structure. Table 2 represents the numerical values of secondary structure content for both proteins.

## 3. Materials and Methods

### 3.1. Enzyme Production and Purification 

Previously described mutant versions of BglA and BglB with increased stability were used in this study: BglA E96K, T385A, N411S, M416I, and N437K [22]; and BglB H62R, N223Y, and M319I [23]. The gene coding sequences were inserted in the *E. coli* expression vector pQE80L (Qiagen) and cloned in the strain Rosetta2 (EMD Millipore). His-tagged protein was purified from transformant clones using nickel-affinity chromatography. The purified protein was stored at 4 °C in a buffer containing Tris-HCl (20 mM) and NaCl (50 mM) at pH 7.2. 

### 3.2. Extrinsic Fluorescence

Fluorescence caused by the binding of the proteins to either ThT or ANS was monitored on a CLARIOstar (BMG, LABTECH GmbH, Ortenberg, Germany) microplate reader with a gain setting of 1500 units under specific pH and temperature conditions. The protein concentration was 1 mgmL^−1^. ThT and ANS were added at a final concentration of 10 μM each in 50 mM sodium phosphate buffer at the different pH values. ThT fluorescence was measured at 450 nm (excitation) and 485 nm (emission), ANS fluorescence was measured at 380 nm (excitation) and 470 nm (emission). All experiments were performed in triplicate in a 96-well black plate with a final volume of 100 μL. Measurements were recorded every 30 min over a total time of 16 h. Temperature was controlled using the same CLARIOstar microplate reader, except in the case of 65 °C, where the 96-well plate was kept in a thermostated incubator.

### 3.3. Activity Assays

β-glucosidase activity was measured using p-nitrophenyl-β-D-glucopyranoside (PNP-Glu) (Merck), as the substrate according to the previously described procedures [23]. For the determination of the residual activity, aliquots of the protein solutions, taken at the indicated time, were added to the reaction medium containing PNP-Glu (5 mM final concentration) in 50 mM phosphate, 10 mM NaCl, 1 mM MgCl_2_, pH 6.5. The reactions were allowed to proceed for 5 min and finished by the addition of Na_2_CO_3_ (1 M). Absorbance was measured at 400 nm. 

### 3.4. Intrinsic Fluorescence

Protein samples were added to pre-warmed tubes at the corresponding temperature, which contained 50 mM sodium phosphate buffer at the corresponding pH (3, 7, or 12). Measurements were performed in a quartz cell using a Hitachi F-2710 fluorescence spectrophotometer (Hitachi, Tokyo, Japan). Excitation wavelengths of 280 nm and 295 nm were used, and the maximum emissions were obtained at 300 nm and 310 nm, respectively. Aliquots were incubated in a heat block and measured at the corresponding times.

### 3.5. Microscopy 

Optical microscopy images were recorded using an Eclipse 90i fluorescence microscope (Nikon Instruments Inc., Melville, N.Y., USA). The fluorescent filter blocks used were UV2-A [EX 330–380, DM 400, LP 420] for THT staining and G-2A [EX 510–560, DM 575, BA 590] for Congo red staining. Confocal images were obtained with a ZEISS LCS 980 with Airyscan 2 (Carl Zeiss Microscopy GmbH, Jena, Germany), a super-resolution confocal microscope.

Electron micrograph images were recorded using a ZEISS ULTRA 55 field-emission scanning electron microscope (FESEM) (FELMI-ZFE, Graz, Austria). The samples (2 μL) were added to a carbon tape and allowed to dry for 24 h. A platinum cover was added before observation.

### 3.6. Circular Dichroism

CD assays were carried out using a Jasco J-815 spectropolarimeter (JASCO, Tsukuba, Japan). Protein samples were diluted in 20 mM phosphate buffer at pH 7 and 25 °C to a final concentration of 0.05 mg/mL. The samples were read at wavelengths of 190–260 nm in a 0.2 cm pitch cell. Measurements were repeated three times. DichroWeb (http://dichroweb.cryst.bbk.ac.uk, accessed on 10 July 2024) was used for the analysis of the spectra with the following settings. Input units: millidegrees/theta; wavelength step (nm): 190; analysis program: CDSSTR; reference set: set 4 (optimized for 190–240 nm); output units: mean residue ellipticity.

## Figures and Tables

**Table 1:** Intrinsic fluorescence, extrinsic fluorescence, and activity of BglA and BglB subjected to different conditions of temperature and pH.

**BglA**									
**Temperature**		**IF 280 nm**	**IF 295 nm**	**ThT**		**ANS**		**Residual** **Activity**	
**°C**	**pH**	**1 h**	**1 h**	**1 h**	**16 h**	**1 h**	**16 h**	**1 h**	**16 h**
25	3	0.6	0.5	0.9	1.4	1	4	0.6	0.9
	7	1	1	1	1.1	1	1.4	1	1
	12	0.7	0.9	0.6	1	1.5	3	0.9	0.9
45	3	0.5	0.8	0.4	0.1	1	1	0.5	0.8
	7	1.2	0.6	0.6	0.5	0.7	0.8	1.2	1
	12	0.2	0.7	0	0	1.5	2.3	0.7	1
65	3	0.4	0.5	0.3	1.5	0.6	1	0	0
	7	0.9	0.3	0.3	1	0.5	0.6	1.1	0
	12	0.15	0.2	0.9	0.8	0.9	0.8	0	0
**BglB**									
**Temperature**		**IF 280 nm**	**IF 295 nm**	**ThT**		**ANS**		**Residual** **Activity**	
**°C**	**pH**	**1 h**	**1 h**	**1 h**	**16 h**	**1 h**	**16 h**	**1 h**	**16 h**
25	3	1.5	0.5	0.9	1.3	1	1.4	0.8	0.7
	7	1	1	1	0.8	1	1	1	1
	12	0.9	0.7	0.6	0	1.5	7.8	0.8	0.6
45	3	0.4	0.8	6.5	24.5	3.8	19.8	0.2	0
	7	1.5	0.6	2.4	10.6	1.2	10.3	0.9	0.6
	12	0.4	0.6	0	0	0.8	2	0	0
65	3	4.2	3.7	3.9	7.9	2.5	4.8	0	0
	7	0.6	0.6	1.2	4.1	1	3.4	0	0
	12	0.6	1.1	0	0	0.3	0.3	0	0

**Table 2:** Protein secondary structure content of BglA and BglB as deduced from the CD spectra recorded after incubation for 16 h at pH 7 or 4.

Protein	pH	α-Helix ^a^	β-Sheet ^a^	Turns ^a^	Unordered ^a^	NRMSD ^b^
BglA	7	28	16	15	25	0.015
	4	3	34	26	31	0.032
BglB	7	31	14	14	24	0.018
	4	7	34	23	30	0.023

^a^ Percentage of different secondary structures. ^b^ NRDMS: Normalized root deviation mean square.

## Data Availability

Data is contained within the article.
